# Gintropy: Gini Index Based Generalization of Entropy

**DOI:** 10.3390/e22080879

**Published:** 2020-08-10

**Authors:** Tamás S. Biró, Zoltán Néda

**Affiliations:** 1 Wigner Research Centre for Physics, 1121 Budapest, Hungary; biro.tamas@wigner.hu; 2 Complexity Science Hub, 1080 Vienna, Austria; 3 Department of Physics, Babeş-Bolyai University, 400084 Cluj-Napoca, Romania

**Keywords:** entropy, Gini index, socio-economic inequalities, econophysics

## Abstract

Entropy is being used in physics, mathematics, informatics and in related areas to describe equilibration, dissipation, maximal probability states and optimal compression of information. The Gini index, on the other hand, is an established measure for social and economical inequalities in a society. In this paper, we explore the mathematical similarities and connections in these two quantities and introduce a new measure that is capable of connecting these two at an interesting analogy level. This supports the idea that a generalization of the Gibbs–Boltzmann–Shannon entropy, based on a transformation of the Lorenz curve, can properly serve in quantifying different aspects of complexity in socio- and econo-physics.

## 1. Introduction

### 1.1. Motivation

Many researchers use entropy as an appropriate measure for quantifying complexity or the inequality level in a complex system. There is an overwhelming choice in generalized entropy formulas, some of them satisfying more of the basic axioms than the others [1]. The classical Boltzmann–Gibbs–Shannon formula is often used in economic and social studies without elaborating too much on the conditions under which it is an appropriate thermodynamic function. Most prominently, the additivity of entropy upon the factorization of probabilities is, as a rule, not tested and therefore the use of entropy remains at the level of a crude analogy. Using the Tsallis- or Rényi entropy formula [2] is also not a sufficient choice. Although a free parameter in this entropy provides more flexibility in processing and interpreting statistical data and generalizing the additivity, there is no basic reason to not use yet another formula that satisfies the basic physical requirements for the entropy.

On the other hand, the most popular way for quantifying the inequality level in a socio-economic system is to use the Gini index, introduced for the first time by the economist Corrado Gini [3]. This measure provides a simple method of quantifying the deviation from a uniform distribution, and it is not a quantity borrowed by a simple analogy from thermodynamics. It also has the advantage that its value is a number in the [0,1] interval, like an order parameter. The Gini index is 0 when all members of the investigated society are equal in the relevant quantity, and it is 1 if one member is monopolizing the whole of the available resources. The Gini index can be determined experimentally either graphically by constructing the Lorentz curve [4], or by the simple formula

(1) $$G=\frac{1}{\langle x\rangle }\frac{\sum_{i=1}^{N}\sum_{j=1}^{N}\left|x_{i}-x_{j}\right|}{2N^{2}},$$

where $x_{i}$ is the relevant quantity for element *i*, and 〈x〉 is its average value for the whole system with *N* elements. While the Gini index is traditionally used to measure wealth-, income- or other inequality, the entropy is a concept stemming from physics and mathematics and is applied to understand, describe and construct optimal or equilibrium distributions. At first glance, these two termini show no reason to be connected. However, in recent publications, it has been observed that the Gini index and the total Shannon entropy of socio-economical models and data show a synergic behavior [5].

In this paper, we shall demonstrate that the mathematical construction formulas of the Gini measure of inequality in a society on the one hand and the entropy–probability trace formula on the other hand bring intriguing similarities at a certain step of their derivation. Both quantities are integrated quantities, in the sense of summing over alternative values of a basic variable, *x*. We propose the usage of the phrase *”gintropy”* in order to express the combination of the Gini index [3,6,7] and the entropy, both associated with a probability density distribution (PDF).

### 1.2. Basics

Let us consider the relevant quantity of the investigated system as a continuous variable *x*. This could be, for example, salary, wealth, population, etc. The occurrence frequency of this given value in a huge set of data are described by the normalized PDF:

(2) $$\int_{0}^{\infty }\;\;\rho \left(x\right)\;dx\;=\;1.$$

An approximation to such mathematical PDFs is given in the praxis by observing the number of occurrences of values in a short bin [x,x+dx] and dividing these by their sum, the total number:

(3) $$\rho \left(x\right)=\lim_{\Delta x\to 0}\frac{N(x,x+\Delta x)}{N_{\mathrm{tot}}\cdot \Delta x}$$

with $N_{\mathrm{tot}}$ the total number of observed data. In income distributions, for example, N(x,x+Δx) is the number of persons having an income in the Δx interval starting at *x*. The total income is then obtained as

(4) $$X_{\mathrm{tot}}\;=N_{tot}\;\int_{0}^{\infty }\;\;x\;\rho \left(x\right)\;dx,$$

and the average income is given by

(5) $$\langle x\rangle \;=\;\int_{0}^{\infty }\;\;x\;\rho \left(x\right)\;dx\;=\;\frac{X_{\mathrm{tot}}}{N_{\mathrm{tot}}}.$$

Both the entropy and the Gini index can be expressed as expectation values of some functions of *x* over the PDF ρ(x), and we are going to demonstrate the latter in the present paper.

Not only the PDFs, but frequently the cumulative distributions are in our light-spot. The first reason for this is that the experimental shape of the cumulative functions are smoother even in the case of a poorer statistics. The second reason is that, especially for income distribution and inequality, the total body of “rich” is better contrasted to the “poor”.

It is straightforward to construct the quantity “the population fraction of richer than *x*” as the tail-cumulative integral of the PDF:

(6) $$\bar{C}\left(x\right)\;=\;\int_{x}^{\infty }\;\;\rho \left(y\right)\;dy.$$

A similar cumulative quantity is the wealth accumulated by this richer class, divided by the average income:

(7) $$\bar{F}\left(x\right)\;=\;\frac{1}{\langle x\rangle }\;\int_{x}^{\infty }\;\;y\;\rho \left(y\right)\;dy.$$

Trivially, one obtains $\bar{C}\left(0\right)=1$ and $\bar{F}\left(0\right)=1$.

The famous Pareto-law expresses that a *p* fraction of the population possesses a (1−p) fraction of the wealth. In the original statement about the economy at the end of 19th century, it was p=0.2, formulated as the “80/20” rule: 20 percent of the population having 80 percent of the total wealth [8,9,10]. Later, a “90/20” rule has also been suggested by Dunford [11]; this loses, however, the elegant definition of the *Pareto point* (see the next paragraph). Analyses of national GDP comparisons and wealth distribution in certain countries often use in the wealthy region a power-law fit, $\rho \left(x\right)=cx^{-(1+\alpha )}$, calling the parameter α the Pareto-index [12,13,14,15]. It is however largely debated where should one consider the cut-off in the distribution curve, over which the tail is of the power-law type. For part of the PDF, exponential fits can also be done [16]. As an overall fit to the whole income distribution curve recently, it has been shown that a Tsallis–Pareto cut power-law or some special beta prime distribution works well [17].

For a simple division of the system in an upper and lower class the $x_{P}$ Pareto-point is used, satisfying:

(8) $$\bar{C}\left(x_{P}\right)\;=\;p,\;\mathrm{while}\;\bar{F}\left(x_{P}\right)\;=\;1-p.$$

The implicit relation, $x_{P}\left(p\right)$, depends on the underlying PDF, ρ(x). Since $\bar{C}\left(0\right)+\bar{F}\left(0\right)=2$ and the general sum is monotonically decreasing, due to

(9) $$\frac{d}{dx}\left(\bar{C}\left(x\right)+\bar{F}\left(x\right)\right)=-\left(1+\frac{x}{\langle x\rangle }\right)\rho \left(x\right)\;\leq \;0,$$

there is always a point $x=x_{P}$, where $\bar{C}\left(x_{P}\right)+\bar{F}\left(x_{P}\right)=1$. However, the value *p* cannot be arbitrary.

As we shall discuss in the next section, the Gini index, *G*, can be expressed in several alternative ways: (i) as the average of big differences in the data set, (ii) as a construction using the above cumulative quantities or (iii) as an expectation value of the cumulative of the cumulative. *G* expressed as an integral over $\bar{C}$ contains an integrand $\sigma (\bar{C})$. For some PDFs, this function turns out to be formally identical with the terms in entropy—a probability trace formula known from elsewhere. These formulas define the *gintropy*, as a function of the cumulative measure of being “richer than”, $\sigma (\bar{C})$—and this function coincides with the classical entropy for an exponential PDF, like the Gibbs–Boltzmann distribution of energy in thermodynamics. For some other, frequently considered distributions in complex systems, the *gintropy* resembles terms of various generalizations of the Gibbs–Boltzmann-Shannon entropy. Among others, we arrive at the Tsallis entropy for the original Pareto distribution, and some further interesting cases. By construction, as we shall demonstrate later, the *gintropy* curve is the difference between the Lorenz curve and the diagonal in the $\bar{F}$ vs. $\bar{C}$ maps.

In the sequel of this paper, we explore these formulas as several facets of the Gini index and its calculation. After the mathematical definitions and equivalent forms, we present certain analytically given PDFs, each reflecting a theoretical possibility about income inequalities: extreme communism giving every person the same income; a divided society defining two classes of the previous case with a fixed share; eco-window, providing equal probability to any income in a fixed, but possibly even an infinite interval; the exponentially distributed income taken as an analogy to the nature of atomic physics; and finally the Pareto-distribution characteristic to capitalism. A different Gini index, *G*, and also a different *gintropy*, $\sigma (\bar{C})$ belong to each model. Finally, we collect a few ideas about what laws the Gini index and *gintropy* may follow: is there a trend akin to the second law of thermodynamics? Are societies closed systems or not? Can or must inflation distort our analysis?

## 2. Gross Inequality in General

Let ρ(x) be a normalized PDF. The Gini index in the continuous *x* case is defined as:

(10) $$G\;\equiv \;\frac{1}{2\langle x\rangle }\;\int_{0}^{\infty }\;\;dx\;\int_{0}^{\infty }\;\;dy\;\left|x-y\right|\;\rho \left(x\right)\;\rho \left(y\right)=\frac{1}{\langle x\rangle }\;\int_{0}^{\infty }\;\;dx\;\int_{x}^{\infty }\;\;dy\;\left(y-x\right)\;\rho \left(x\right)\;\rho \left(y\right).$$

It can easily be proven that its value is always between zero and one, and is used to quantify the gross inequality in the distribution ρ(x). The original definition (10) can be expressed by using the cumulatives as

(11) $$G\;=\;\int_{0}^{\infty }\;\;\;\rho \left(x\right)\;\left[\bar{F}\left(x\right)\;-\;\frac{x}{\langle x\rangle }\;\bar{C}\left(x\right)\right]dx.$$

This expression can be further comprised by considering the cumulative of the cumulative:

(12) $$\bar{h}\left(x\right)\;\equiv \;\int_{x}^{\infty }\;\;dy\;\bar{C}\left(y\right)\;=\;\int_{x}^{\infty }\;\;dy\int_{y}^{\infty }\;\;dz\;\rho \left(z\right)\;=\;\int_{x}^{\infty }\;\;dz\int_{x}^{z}\;\;dy\;\rho \left(z\right)\;=\;\int_{x}^{\infty }\;\;\left(z-x\right)\rho \left(z\right)\;dz=\langle x\rangle \bar{F}\left(x\right)-x\bar{C}\left(x\right).$$

Finally, from here, the Gini index is then expressed as a ratio of two expectation values:

(13) $$G\;=\;\frac{1}{\langle x\rangle }\int_{0}^{\infty }\;\;\rho \left(x\right)\;\bar{h}\left(x\right)\;dx\;=\;\frac{\langle \bar{h}\left(x\right)\rangle }{\langle x\rangle }.$$

Alternatively, it can be solely expressed via the cumulative population. From the corresponding definitions, we have the derivatives: $\rho \left(x\right)=-d\bar{C}/dx$, $x\rho \left(x\right)=-\langle x\rangle d\bar{F}/dx$ and therefore $x=\langle x\rangle d\bar{F}/d\bar{C}$. Using the immediate equations, $\rho \left(x\right)=\frac{d^{2}\bar{h}\left(x\right)}{dx^{2}}$ and $\bar{C}\left(x\right)=-\frac{d\bar{h}\left(x\right)}{dx}$, from Equation (13), and, integrating by parts, we get:

(14) $$\langle x\rangle \;G\;=\;\int_{0}^{\infty }\;\;\bar{h}\;\frac{d^{2}\bar{h}}{dx^{2}}\;dx\;=\;\bar{h}\left(0\right)\bar{C}\left(0\right)-\int_{0}^{\infty }\;\;{\bar{C}}^{2}\left(x\right)\;dx.$$

Using the boundary conditions $\bar{C}\left(0\right)=1$ and $\bar{h}\left(0\right)=\langle x\rangle$, we arrive at

(15) $$G\;=\;1\;-\;\frac{1}{\langle x\rangle }\int_{0}^{\infty }\;\;{\bar{C}}^{2}\left(x\right)\;dx\;=\;\frac{1}{\langle x\rangle }\int_{0}^{\infty }\;\;\bar{C}\;\left(1-\bar{C}\right)\;dx.$$

This form is reminiscent of the quantum impurity measure, $Tr(\rho -\rho^{2})$, which is zero only for pure states. In the theory of searching trees in informatics, the expression $I_{G}=\sum_{i}\left(p_{i}-p_{i}^{2}\right)$ is called Gini impurity measure [18]. Let us also note here that for scaling PDFs, i.e., $\rho \left(x\right)=\frac{1}{\langle x\rangle }\;f\left(\frac{x}{\langle x\rangle }\right)$, the cumulative functions, $\bar{C}$, and the Gini index, *G*, do not depend directly on $\langle x\rangle$, it depends only on the form of the f(z) function. This is important when studying the history (time evolution) of *G* and the related constructions: an overall inflation increasing $\langle x\rangle$ in time will not influence this inequality measure.

**Figure 1:**
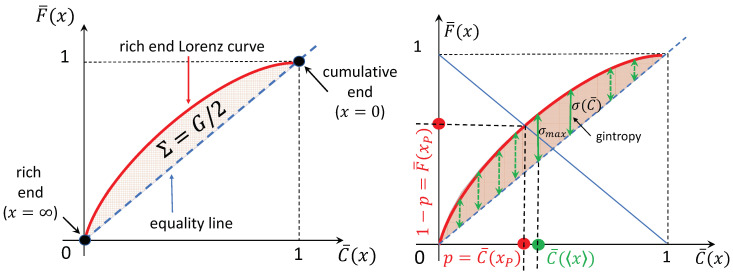
(**left**) The rich end Lorenz curve, and connection with the Gini index. (**right**) Visual illustration of the gintropy, the Pareto Point and the maximal gintropy.

Finally, we arrive now at the construction of the quantity *gintropy*. A fashionable representation of the Gini index is realized by plotting the cumulative wealth percentage in terms of the cumulative population possessing that wealth like in Figure 1. It can be shown that the half-moon area between the Lorenz curve [4,19,20,21,22] and the diagonal of the unit square (known as *equality line*) in such an $\bar{F}\left(x\right)$ vs. $\bar{C}\left(x\right)$ plot,

(16) $$\Sigma \;\equiv \;\int_{0}^{1}\;\;\sigma \left(\bar{C}\right)\;d\bar{C},$$

is exactly G/2. The integrand, $\sigma (\bar{C})$, under the integral over $\bar{C}$—which runs between zero and one—behaves alike an entropy-density. (The original and nowadays used Lorenz curve actually maps the low-cumulatives, integrated from zero to *x*. However, σ(C) instead of $\sigma (\bar{C})$ does not remind to entropy formulas). We call this quantity **gintropy**, and define as the difference between the rich-end-cumulative Lorenz curve and the diagonal:

(17) $$\sigma \left(x\right)\;\equiv \;\bar{F}\left(x\right)\;-\;\bar{C}\left(x\right)\;=\;\int_{x}^{\infty }\;\;\left(\frac{y}{\langle x\rangle }-1\right)\;\rho \left(y\right)dy.$$

From the above definition, $\sigma (\bar{C})$ remains to be reconstructed with the help of $\bar{C}\left(x\right)$. We note that, using the relations $\bar{C}\left(x\right)=1-C\left(x\right)$ and $\bar{F}\left(x\right)=1-F\left(x\right)$, this quantity equivalently can be expressed by the poor-end-cumulative Lorenz curve (the generally used form of the Lorenz curve) too:

(18) $$\sigma \left(x\right)\;=\;C\left(x\right)\;-\;F\left(x\right)\;=\;\int_{0}^{x}\;\;\left(1-\frac{y}{\langle x\rangle }\right)\;\rho \left(y\right)dy.$$

The Gini index is expressed from the gintropy as a simple integral

(19) $$G\;=\;2\;\Sigma \;=\;2\int_{0}^{1}\;\;\;\sigma \left(\bar{C}\right)\;d\bar{C}\;=\;2\;\int_{0}^{\infty }\;\;\sigma \left(x\right)\rho \left(x\right)\;dx\;=\;2\langle \sigma (x)\rangle .$$

We note here that, for any integral, one substitutes $\int_{0}^{1}\;\;f\left(\bar{C}\right)\;d\bar{C}\;=\;\int_{0}^{\infty }\;\;f\left(\bar{C}\left(x\right)\right)\;\rho \left(x\right)\;dx$.

It is interesting to summarize the proof of this statement here because it is a central motivation of thinking in terms of gintropy. Using the respective definitions of the tail-cumulative quantities, the half-moon area (16) is calculated as the following double integral:

(20) $$\Sigma \;=\;\int_{0}^{\infty }\;\;dx\;\rho \left(x\right)\;\int_{x}^{\infty }\;\;dy\;\left(\frac{y}{\langle x\rangle }-1\right)\;\rho \left(y\right).$$

Changing the order of integration leads to

(21) $$\Sigma \;=\;\int_{0}^{\infty }\;\;dy\int_{0}^{y}\;\;dx\;\rho \left(x\right)\left(\frac{y}{\langle x\rangle }-1\right)\;\rho \left(y\right)\;=\;\int_{0}^{\infty }\;\;dy\;\left(1-\bar{C}\left(y\right)\right)\left(\frac{y}{\langle x\rangle }-1\right)\;\rho \left(y\right).$$

Here, the term with 1 in the first parenthesis integrates to zero due to the definition of the expectation value, $\langle x\rangle$. Then, we replace $-\bar{C}\left(y\right)\rho \left(y\right)=\frac{1}{2}\frac{d}{dy}{\bar{C}}^{2}$, integrate by parts and compare the result to (15) to conclude:

(22) $$\Sigma \;=\;\;\frac{1}{2}{\bar{C}}^{2}\left(0\right)-\frac{1}{2\langle x\rangle }\int_{0}^{\infty }\;\;dy\;{\bar{C}}^{2}\left(y\right)\;=\;\frac{1}{2}\;G.$$

Now, we explore some basic properties of gintropy. Some of these provide further evidence to consider gintropy like a generalized entropy density.

1. The gintropy is never negative: $\sigma =\bar{F}-\bar{C}\geq 0$ is proven by inspecting the integral
  $$\sigma \left(x\right)\;=\;\int_{x}^{\infty }\;\;\left(y/\langle x\rangle -1\right)\rho \left(y\right)dy\;=\;\int_{0}^{x}\;\;\left(1-y/\langle x\rangle \right)\rho \left(y\right)dy\;\geq \;0,$$
  and taking the first form for $x\geq \langle x\rangle$, the second form for the opposite case. This implies that the rich-end wealth fraction is always bigger or equal to the population fraction possessing it.
2. The gintropy is maximal at $x=\langle x\rangle$, $\sigma_{max}=\sigma \left(\langle x\rangle \right)$, since $d\sigma /dx=\left(1-x/\langle x\rangle \right)\rho \left(x\right)$ changes its sign exactly there and only there.
3. According to Equation (8) at the Pareto-point, the gintropy equals $\sigma (x_{P})=1-2p$, and therefore, for the Pareto point, p≤1/2 holds for the rich fraction. Since $\sigma_{max}\geq \sigma \left(x_{P}\right)$, in order to get a Pareto point: σ(〈x〉)≥1−2p, i.e., the maximum of the gintropy has to be bigger than this difference value. As a consequence for the Pareto Point, we have a restriction imposed by the maximal gintropy $(1-\sigma \left(\langle x\rangle \right))/2\leq p\leq 1/2$.
4. The expectation value of gintropy is the half of the gini index: $\int_{0}^{\infty }\;\;\sigma \left(x\right)\;\rho \left(x\right)\;dx\;=\;\int_{0}^{1}\;\;\sigma \left(\bar{C}\right)\;d\bar{C}\;=\;\Sigma \;=\;G/2$.
5. The integral of gintropy over the base value *x* is the non-Poissonity index, $\int_{0}^{\infty }\;\;\sigma \left(x\right)\;dx=\frac{\mathrm{Var}(x)}{\langle x\rangle }$, with $\mathrm{Var}\left(x\right)=\langle x^{2}\rangle -{\langle x\rangle }^{2}$ being the variance of *x*. The proof of this statement uses the same mathematical trick as the one in Equation (12).
6. For some particular PDFs, $\sigma (\bar{C})$ looks like an entropy density formula, $s(p_{i})$. We present important examples in the next section.

## 3. Important Examples

In this section, we list some important examples of the gintropy, $\sigma (\bar{C})$. We go through primitive models of income/wealth distributions, labelled as communism, communism++, eco-window, natural, or capitalism. Starting from model PDFs, the gintropy expression and the Gini index are calculated.

### 3.1. Communism

Our first example is communism: all incomes are equal, the PDF is simply a singular delta-distribution, peaked at the single value *a*: ρ(x)=δ(x−a) leading to $\langle x\rangle =a$, $\bar{C}\left(x\right)=\Theta \left(a-x\right)$ and $\bar{h}\left(x\right)=\left(a-x\right)\Theta \left(a-x\right)$, with Θ(x) the Heaviside step function defined as:

(23) $$\Theta \left(x\right)\;=\;\left\{\begin{array}{c} 0\;x<0\\ \frac{1}{2}\;x=0\\ 1\;(x>0) \end{array}\right.$$

This leads to $\langle x\rangle \bar{F}=\bar{h}+x\bar{C}=a\Theta \left(a-x\right)$ and by that

(24) $$\sigma \left(x\right)\;=\;\bar{F}\left(x\right)\;-\;\bar{C}\left(x\right)\;=\;0,$$

i.e., to an identically vanishing gintropy. As a consequence, also G=0. Here, the Pareto-point belongs to a 50/50 division.

### 3.2. Communism++

The next example we present is a slight variation of the previous: now, two peaks in a given ratio constitute the PDF. This belongs to a two-class-society where *all are equal but some of them are more equal*. The two-peak-PDF, ρ(x)=wδ(x−a)+(1−w)δ(x−b) (b>a), delivers $\langle x\rangle =w\;a+\left(1-w\right)\;b$. The *w* fraction of the population has an income *a* and the (1−w) fraction *b*. The cumulative rich population graph shows two steps, at *a* and *b*, respectively:

(25) $$\bar{C}\left(x\right)\;=\;w\;\Theta \left(a-x\right)+\left(1-w\right)\;\Theta \left(b-x\right),$$

having the value 1 for x≤a, (1−w) for x∈[a,b], and 0, otherwise. Therefore, $C\left(x\right)=1-\bar{C}\left(x\right)$ is zero for x≤a, equals *w* in the mid interval, and has the value 1 otherwise. The Gini index is obtained from this as:

(26) $$G\;=\;\frac{1}{\langle x\rangle }\int_{0}^{\infty }\;\;\;\bar{C}\left(1-\bar{C}\right)dx\;=\;\frac{1}{\langle x\rangle }\;\left(b-a\right)\;w\;\left(1-w\right).$$

Expressing the weights, $w=\frac{b-\langle x\rangle }{b-a}$ and $1-w=\frac{\langle x\rangle -a}{b-a}$, we obtain the alternative form

(27) $$G\;=\;\frac{\left(\langle x\rangle -a\right)\left(b-\langle x\rangle \right)}{\left(b-a\right)\langle x\rangle }.$$

It is worth noting that, for a→0, i.e., when the lower class has (almost) zero income, the Gini index, cf. (26) tends to G→w, exactly the share of the people earning a→0 in the population. This result is independent of *b*, the income in the upper class.

The gintropy, following its definition, first is expressed as a function of *x*:

(28) $$\sigma \left(x\right)\;=\;\bar{F}\left(x\right)\;-\;\bar{C}\left(x\right)\;=\;w\left(\frac{a}{\langle x\rangle }-1\right)\Theta \left(a-x\right)\;+\;\left(1-w\right)\left(\frac{b}{\langle x\rangle }-1\right)\Theta \left(b-x\right).$$

It is easy to see that, outside the interval [a,b], the gintropy is zero. Inside the interval, only the second term survives giving

(29) $$\sigma \left(x\right)\;=\;G\;\left[\Theta (b-x)-\Theta (a-x)\right].$$

In conclusion, $\sigma (\bar{C})$ shows a plateau at $\bar{C}=1-w$ with the value *G* and its jumps are at $\bar{C}\left(a\right)=1-w/2$ and $\bar{C}\left(b\right)=\left(1-w\right)/2$:

(30) $$\sigma \left(\bar{C}\right)\;=\;G\;\left[\Theta \left(\bar{C}\left(a\right)-\bar{C}\right)-\Theta \left(\bar{C}\left(b\right)-\bar{C}\right)\right].$$

It is easy to check that indeed

(31) $$\Sigma \;=\;\int_{0}^{1}\;\;\sigma \left(\bar{C}\right)\;d\bar{C}\;=\;G\left[\bar{C}\left(a\right)-\bar{C}\left(b\right)\right]\;=\;G/2.$$

**Figure 2:**
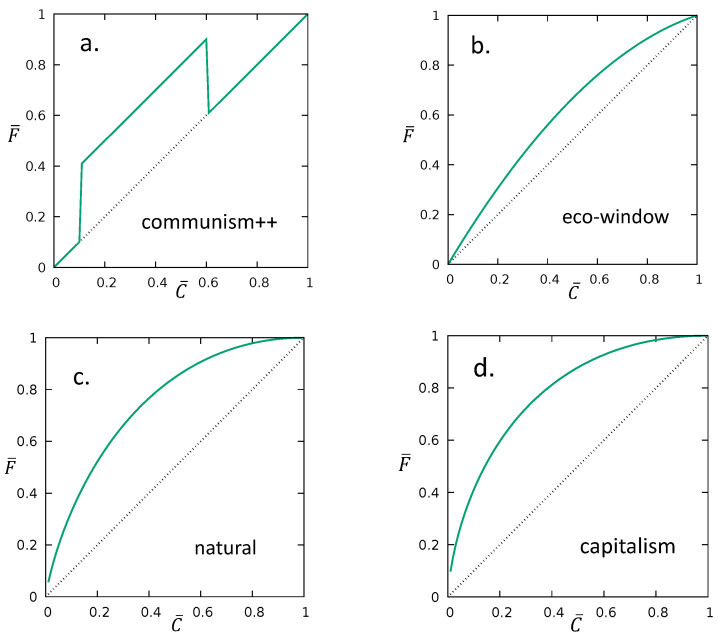
The $\bar{F}$ vs. $\bar{C}$ cumulative maps (Lorenz curves) for the (**a**) communism++ (a=1, b=4 and w=0.8), (**b**) eco-window (a=1,b=5), (**c**) natural exponential ($\langle x\rangle =1$) and for the capitalism (**d**) (A=1,B=3→q=3/4) distributions. The corresponding Gini index are G=0.3, G=2/9, G=1/2 and G=4/7, respectively.

The corresponding Lorenz curve is illustrated in Figure 2a.

### 3.3. Eco-Window

The next example is still mathematically simple with a window-form PDF. We label this as *eco-window*: here, everyone has the same chance for all of possible incomes between *a* and *b*. Eventually, a=0 and/or b=∞ may be considered, as special cases. For the PDF $\rho \left(x\right)\;=\;\frac{1}{b-a}\;\left[\Theta \left(b-x\right)-\Theta \left(a-x\right)\right]$, one obtains the following cumulative rich distribution:

(32) $$\bar{C}\left(x\right)\;=\frac{b-x}{b-a}\Theta \left(b-x\right)-\frac{a-x}{b-a}\Theta \left(a-x\right)\;=\left\{\begin{array}{c} 1\;(x<a)\\ \frac{b-x}{b-a}\;x\in [a,b]\\ 0\;(x>b) \end{array}\right.$$

Obviously, $\langle x\rangle =\left(a+b\right)/2$ and, according to Equation (15), the Gini index becomes:

(33) $$G\;=\;\frac{1}{\langle x\rangle }\int_{a}^{b}\;\;\frac{\left(b-x)(x-a\right)}{{\left(b-a\right)}^{2}}dx\;=\;\frac{1}{3}\;\frac{b-a}{b+a}.$$

After some tedious but straightforward calculation, the *gintropy* is obtained as a function of $\bar{C}$:

(34) $$\sigma \left(\bar{C}\right)\;=\;3\;G\;\bar{C}\left(1-\bar{C}\right).$$

For a specific choice of *a* and *b*, the corresponding Lorenz curve is illustrated in Figure 2b.

### 3.4. Natural Distribution

Our next example is the natural distribution, mimicking the Boltzmann–Gibbs exponential energy distribution, known from statistical physics. This is not necessarily an equilibrium distribution, it may also be the stationary limit of “growth and resetting” type processes with quantity-independent rates [23]. The PDF is a scaling one: $\rho \left(x\right)\;=\;\frac{1}{\langle x\rangle }e^{-x/\langle x\rangle }$. The corresponding tail-cumulative probability, the rich population is given by

(35) $$\bar{C}\left(x\right)\;=\;e^{-x/\langle x\rangle },$$

and the Gini index becomes

(36) $$G\;=\;1\;-\;\frac{1}{\langle x\rangle }\int_{0}^{\infty }\;\;e^{-2x/\langle x\rangle }dx\;=\;\frac{1}{2}.$$

Our gintropy formula is constructed as follows: first, we obtain the cumulative of the cumulative,

(37) $$\bar{h}\;=\;\int_{x}^{\infty }\;\;e^{-y/\langle x\rangle }\;dy\;=\;\langle x\rangle \;e^{-x/\langle x\rangle }.$$

From this, it is easy to obtain the wealth share of the rich classes, $\langle x\rangle \bar{F}=\bar{h}+x\bar{C}=\left(x+\langle x\rangle \right)e^{-x/\langle x\rangle }$, and based on this the gintropy

(38) $$\sigma \left(x\right)=\frac{x}{\langle x\rangle }e^{-x/\langle x\rangle }.$$

In order to express it as a function of $\bar{C}$, we invert (35) to have

(39) $$x\left(\bar{C}\right)\;=\;-\langle x\rangle \;\ln\bar{C}.$$

Finally, it leads to

(40) $$\sigma \left(\bar{C}\right)\;=\;-\bar{C}\;\ln\bar{C}.$$

Apart from a constant proportionality factor, this formula formally coincides with the terms in the sum of the Boltzmann–Gibbs–Shannon entropy:

(41) $$S=-k\sum_{i}p_{i}\ln\left(p_{i}\right)$$

To continue the analogy, also $\bar{C}\in \left[0,1\right]$. Indeed, in this case, gintropy is like the entropy density, with the caveat that the cumulative values $\bar{C}\left(x\right)$ are never disjunct for different *x*-s; instead, they overlap and show a definite hierarchy. The Lorenz curve for 〈x〉=1 is illustrated in Figure 2c.

### 3.5. Capitalism

Our last example is capitalism, conjecturing the base PDF being the cut Pareto (known also as Tsallis–Pareto or Lomax II) distribution [24]:

(42) $$\rho \left(x\right)=A\left(B+1\right){\left(1+Ax\right)}^{-B-2}$$

This distribution can also be obtained as the canonical equilibrium optimizer of the Tsallis entropy [25].

The tail-cumulative integral is

(43) $$\bar{C}\left(x\right)={\left(1+Ax\right)}^{-B-1},$$

which upon integration leads to the following cumulative of the cumulative:

(44) $$\bar{h}\left(x\right)=\frac{1}{AB}{\left(1+Ax\right)}^{-B}.$$

This result also delivers the expectation value, $\langle x\rangle =\bar{h}\left(0\right)=1/AB$. The Gini index is calculated in the (15) form, and it becomes

(45) $$G\;=\;1-AB\int_{0}^{\infty }\;\;{\left(1+Ax\right)}^{-2B-2}dx\;=\;\frac{B+1}{2B+1}.$$

The gintropy as a function of the income, *x*, follows the form

(46) $$\sigma \left(x\right)\;=\;A\left(B+1\right)\;x\;{\left(1+Ax\right)}^{-B-1}.$$

In order to express this result akin to the entropy, we write σ as a function of $\bar{C}$ using the inversion of Equation (43)

(47) $$x\left(\bar{C}\right)\;=\;\frac{1}{A}\left({\bar{C}}^{\;-\frac{1}{B+1}}\;-\;1\right),$$

and we obtain

(48) $$\sigma \left(\bar{C}\right)\;=\;\left(B+1\right)\;\left({\bar{C}}^{\frac{B}{B+1}}\;-\;\bar{C}\right).$$

Finally, using the Tsallis parameter, q=B/(B+1), we arrive at the formula:

(49) $$\sigma \left(\bar{C}\right)=\frac{1}{1-q}\left({\bar{C}}^{q}-\bar{C}\right),$$

One immediately makes an analogy with the terms in the Tsallis entropy formula:

(50) $$S_{q}=\frac{k}{1-q}\sum_{i}\left(p_{i}^{q}-p_{i}\right),$$

The Gini index is simply

(51) $$G\;=\;\frac{1}{q+1}.$$

Similar to the previously considered cases, we illustrate the Lorenz curve for this distributions as well. For A=1 and B=3, the corresponding Lorenz curve is plotted in Figure 2d.

**Figure 3:**
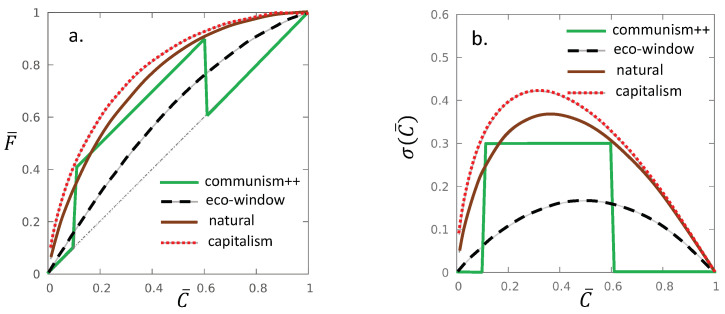
(**a**) $\bar{F}$ – $\bar{C}$ Lorenz curves in one comparison and (**b**) the corresponding **gintropy** curves, $\sigma (\bar{C})$, for the communism++, eco-window, natural and capitalism models. The Gini indices are G=0.3, G=2/9, G=1/2 and G=4/7, respectively.

Finally, we summarize the lesson of the considered theoretical examples in Table 1 and Figure 3.

## 4. Conclusions

In this work, we explored a density-like quantity called *gintropy* which occurs in calculating the Gini index, *G*, for a given relevant socio-economic distribution, ρ(x). This gintropy can be deduced from two cumulative functions, the rich population fraction and the corresponding richness fraction, $\bar{C}\left(x\right)$ and $\bar{F}\left(x\right)$, respectively. The proposed ”gintropy” name is meant to suggest a connection between the inequality measure quantified by the Gini index and the entropy. Its dependence on the rich population fraction cumulative function is reminiscent of terms in entropy formulas, known from physics, statistics, and informatics. More precisely, we found that, for the the natural, exponential PDF, the gintropy is reminiscent of the classical Boltzmann–Gibbs–Shannon formula, $\sigma \left(\bar{C}\right)=-\bar{C}\ln\bar{C}$. The Gini index is then the expectation value of the gintropy function; for the exponential PDF, its value is 1/2. For the Tsallis–Pareto distribution, the Gini index must be always over this value.

Several other PDFs have been suggested to describe income or wealth distributions in terms of time [17,26,27,28,29]. Many of them are not treatable analytically, so the $\sigma (\bar{C})$ relation can only be explored numerically.

Beyond igniting the theoretical phantasy, the gintropy—reminscent of generalized entropy formulas—is also the one-variable density, which lays under the Gini index, originally defined for measuring inequality. Turning this statement around, should we look for such generalizations of the classical entropy formula that are inequality or impurity measures at the same time? We believe that this criterion selects a subclass of possible statistical theories among all possible approaches to the origin, behavior and future of social and economical inequalities. Even generalizations of the Gini index formula have been suggested a few times, cf. [30,31]. We do not expect that a corresponding gintropy (”Lorenz curve minus the diagonal”) would resemble any known entropy formula, but this question needs further study.

Finally, it seems that the “correct” entropy measure for economical and social theories can hardly be a simple copy of the classical formula known from physics, mathematics and informatics. Our procedure, described above, is more promising: a recipe for constructing gintropy from cumulative functions of the underlying PDF whose expectation value is the half Gini index and whose dependency on the cumulative rich population coincides with various generalizations of the entropy–probability formula.

## Figures and Tables

**Table 1:** Summary of PDFs, the gintropy formulas and Gini index values for some ideal income/wealth distribution schemes.

	ρ(x)	$\sigma (\bar{C})$	G
natural	$\frac{1}{\langle x\rangle }e^{-x/\langle x\rangle }$	$-\bar{C}\ln\bar{C}$	$\frac{1}{2}$
capitalism	$\frac{A}{1-q}{\left(1+Ax\right)}^{\frac{-1}{1-q}}$	$\frac{1}{1-q}\left({\bar{C}}^{q}-\bar{C}\right)$	$\frac{1}{q+1}\geq \frac{1}{2}$
eco-window	$\frac{1}{b-a}\left[\Theta \left(b-x\right)-\Theta \left(a-x\right)\right]$	$3G\;\bar{C}\left(1-\bar{C}\right)$	$\frac{1}{3}\;\frac{b-a}{b+a}\leq \frac{1}{3}$
communism++	wδ(x−a)+(1−w)δ(x−b)	$G\;[\Theta \left(\bar{C}\left(a\right)-\bar{C}\right)-\Theta \left(\bar{C}\left(b\right)-\bar{C}\right)]$	$\frac{\left(b-a)q\;(1-w\right)}{w\;a+(1-w)\;b}$
communism	δ(x−a)	0	0
